# Carnosol Inhibits Pro-Inflammatory and Catabolic Mediators of Cartilage Breakdown in Human Osteoarthritic Chondrocytes and Mediates Cross-Talk between Subchondral Bone Osteoblasts and Chondrocytes

**DOI:** 10.1371/journal.pone.0136118

**Published:** 2015-08-20

**Authors:** Christelle Sanchez, Marie-Noëlle Horcajada, Fanny Membrez Scalfo, Laurent Ameye, Elizabeth Offord, Yves Henrotin

**Affiliations:** 1 Bone and Cartilage Research Unit, Arthropôle Liège, University of Liège, Liège, Belgium; 2 Nutrition and Health, Nestle Research Center, Vers- chez- les- Blanc, Lausanne, Switzerland; 3 Physical Therapy and Rehabilitation Department, Princess Paola Hospital, Marche-en-Famenne, Belgium; SERGAS (Servizo Galego de Saude) and IDIS (Instituto de Investigación Sanitaria de Santiago), the NEIRID Lab, Research Laboratory 9, Santiago University Clinical Hospital. Santiago de Compostela, SPAIN, SPAIN

## Abstract

**Aim:**

The aim of this work was to evaluate the effects of carnosol, a rosemary polyphenol, on pro-inflammatory and catabolic mediators of cartilage breakdown in chondrocytes and via bone-cartilage crosstalk.

**Materials and Methods:**

Osteoarthritic (OA) human chondrocytes were cultured in alginate beads for 4 days in presence or absence of carnosol (6 nM to 9 μM). The production of aggrecan, matrix metalloproteinase (MMP)-3, tissue inhibitor of metalloproteinase (TIMP)-1, interleukin (IL)-6 and nitric oxide (NO) and the expression of type II collagen and ADAMTS-4 and -5 were analyzed. Human osteoblasts from sclerotic (SC) or non-sclerotic (NSC) subchondral bone were cultured for 3 days in presence or absence of carnosol before co-culture with chondrocytes. Chondrocyte gene expression was analyzed after 4 days of co-culture.

**Results:**

In chondrocytes, type II collagen expression was significantly enhanced in the presence of 3 μM carnosol (p = 0.008). MMP-3, IL-6, NO production and ADAMTS-4 expression were down-regulated in a concentration-dependent manner by carnosol (p<0.01). TIMP-1 production was slightly increased at 3 μM (p = 0.02) and ADAMTS-5 expression was decreased from 0.2 to 9 μM carnosol (p<0.05). IL-6 and PGE_2_ production was reduced in the presence of carnosol in both SC and NSC osteoblasts while alkaline phosphatase activity was not changed. In co-culture experiments preincubation of NSC and SC osteoblasts wih carnosol resulted in similar effects to incubation with anti-IL-6 antibody, namely a significant increase in aggrecan and decrease in MMP-3, ADAMTS-4 and -5 gene expression by chondrocytes.

**Conclusions:**

Carnosol showed potent inhibition of pro-inflammatory and catabolic mediators of cartilage breakdown in chondrocytes. Inhibition of matrix degradation and enhancement of formation was observed in chondrocytes cocultured with subchondral osteoblasts preincubated with carnosol indicating a cross-talk between these two cellular compartments, potentially mediated via inhibition of IL-6 in osteoblasts as similar results were obtained with anti-IL-6 antibody.

## Introduction

The main feature of osteoarthritis (OA) is the progressive degradation and loss of the articular cartilage accompanied by other critical structural changes, such as synovial membrane inflammation, subchondral bone sclerosis, osteophytes formation at the joint margin, ligament laxity and muscle atrophy [1]. All these structural changes contribute to symptoms of OA (severe pain, stiffness, loss of joint mobility and disability).

Certain cytokines, such as IL-1β and TNF-α produced by activated synoviocytes, chondrocytes or monocytes, play a major role in the onset and the progression of OA [2]. These cytokines stimulate their own production in a paracrine or autocrine manner and induce the production of a wide range of other pro- inflammatory mediators, such as IL-6, IL-8, IL-17, IL-18 and oncostatin M as well as reactive oxygen species such as nitric oxide, superoxide, hydrogen peroxide and peroxynitrite by joint cells [3]. These factors, together with inflammatory prostaglandins (such as PGE_2_), leukotrienes and some adipokines, promote cartilage destruction by increasing the production and secretion of proteinases such as matrix metalloproteinases (MMP) and aggrecanases [A Disintegrin and Metalloproteinase with Thrombospondin Motifs (ADAMTS)-4 and -5)] [2].

Due to the presence of microcracks, vascular channels and neovascularization linking the subchondral bone tissue and cartilage, it is hypothesized that cross-talk may occur between the two tissues. Thus expression of proinflammatory mediators such as IL-6, TGF-β1 and probably some other unexplored factors produced by subchondral osteoblasts could also contribute to the abnormal remodelling of OA cartilage [4, 5].

We have previously demonstrated that osteoblasts isolated from subchondral OA bone expressed an altered phenotype. More precisely, we demonstrated that osteoblasts coming from the thickening (called sclerotic area, SC) of subchondral bone located just below a cartilage lesion exhibits an elevated alkaline phosphatase activity and express higher levels of IL-6, IL-8, PGE_2_, TGF-β1 and type I collagen than osteoblasts coming from the non-thickening neighbouring area (called non-sclerotic area, NSC) [5]. To investigate osteoblasts/chondrocytes crosstalk, we developed an original co-culture model, in which human OA subchondral osteoblasts in monolayer are cultured with human OA chondrocytes in alginate beads. Using this co-culture model, we have previously shown that SC osteoblasts, but not NSC osteoblasts, induced a decrease of aggrecan and type II collagen mRNA levels and an increase of MMP-3 mRNA in chondrocytes [6, 7].

The aim of this study was to assess the chondroprotective effects of carnosol and related mechanisms of action on cartilage breakdown in this co-culture model of chondrocytes and osteoblasts. Carnosol is an anti-inflammatory and anti-oxidant compound from rosemary. Rosemary polyphenols, in particular carnosol and carnosic acid, are among the most potent natural antioxidants, used in food preservation but also with health benefits such as anti-inflammation and detoxification [8]. Carnosol was largely investigated for anti-tumoral, anti-inflammatory and anti-oxidant activities in several cell line and animal models [9–12], but no study was yet dedicated to its potential effect on joint cells and bone. Carnosol suppresses inducible nitric oxide synthase down-regulating nuclear factor-B in mouse macrophages [13]. It suppresses cyclooxygenase (COX)-2 transcription in human mammary epithelial cells [14]. Carnosol also induces Nrf-2, an important transcriptional regulator of antioxidant, anti-inflammatory and detoxification processes [15]. Furthermore, using neutralizing monoclonal antibodies, we have highlighted the role played by mediators modified by carnosol in this osteoblast/chondrocyte crosstalk.

## Materials and Methods

### Origin of cells and ethics statement

OA human articular cartilage and subchondral bone was removed from knee joints patients undergoing total knee-replacement surgery. All subjects provided written informed consent, and ethical approval (ethics Committee of the University of Liège (reference number 2005/8) was granted for this study.

### Chondrocytes culture in alginate beads

OA cartilage was obtained from the knees of 7 men and 2 women aged from 52 to 76 years old, being excised from the superficial and medium layers of cartilage and avoiding the calcified layer, enzymatically digested and primary chondrocytes were cultured in alginate bead at a density of 4 x 10^6^ cells/ml in 1.2% low viscosity alginate (Sigma-Aldrich, Belgium) as previously described [16]. Ten alginate beads containing OA chondrocytes were placed in porous inserts (with 1 μm pore size; Falcon, BD Biosciences, Erembodegem, Belgium). The chondrocytes in alginate beads were maintained two days in DMEM supplemented with 2% Ultroser G, 10 mM HEPES, penicillin (100 U/ml) and streptomycin (100 μg/ml), to avoid contamination with drugs that donors might have taken before death. After this wash out period, culture medium was changed and the chondrocytes were either co-cultured with NSC or SC subchondral osteoblasts for 4 days (see next paragraph), or cultured alone (mono-culture) with carnosol (Cayman Chemical) from 6 nM to 9 μM for 4 days. The experiment on chondrocytes mono-cultures were repeated three times, each time using cells coming from a different donor. Carnosol was first dissolved in DMSO and then diluted in incubation medium to the required final concentration. The final concentration of DMSO was 0.02% in all culture conditions. The nutrient media used was DMEM supplemented with 1% ITS+ (Lonza, Belgium), 10 mM HEPES, 100 U/ml penicillin, 100 μg/ml streptomycin, 2mM glutamine (Lonza, Belgium), 50 μg/ml ascorbic acid (Sigma-Aldrich, Belgium), 20 μg/ml proline (Invitrogen, Belgium). ITS+ is a premixed cell growth system containing in one ml: 0.625 mg insulin, 0.625 mg transferrin, 0.625 μg selenious acid, 0.125 g bovine serum albumin and 0.535 mg linoleic acid.

### Subchondral osteoarthritic osteoblasts in monolayer culture

Tibial subchondral bone plates were obtained from 9 OA men (age ranged from 40 to 79 years). After careful elimination of trabecular bone and articular cartilage, OA subchondral bone was dissected to separate non sclerotic (NSC) from SC zones [5]. We have considered as SC bone only the subchondral bone zones with a thickness greater than 2 mm and either denuded or overlaid by fibrillated cartilage. Also, we have considered as NSC bone only the subchondral bone zones with a maximal thickness of 1 mm. Osteoblasts from SC or NSC subchondral bone were then obtained by outgrowth from explants as previously described [5]. At confluence, primary cells were collected by trypsinization, seeded (50,000 cells/cm^2^) in 12-well plates (12-well companion plates, Falcon, BD Biosciences, Erembodegem, Belgium) and grown for 3 days in DMEM containing 10% FBS, 100 U/ml penicillin, 100 μg/ml streptomycin, 10 mM HEPES. After washings, osteoblasts were maintained for 12 days in a differentiation media, composed of DMEM containing 100 U/ml penicillin, 100 μg/ml streptomycin, 10 mM HEPES, 2% Ultroser G, a serum substitute, 10^−8^ M 1,25(OH)_2_vitaminD_3_ (Sigma-Aldrich, Belgium), 2 mM glutamine, 50 μg/ml ascorbic acid and 20 μg/ml proline. At the end of this differentiation period, cells expressed an osteoblastic phenotype characterized by the production of osteocalcin and alkaline phosphatase [5]. After washings, cells were then cultured in ITS+ medium for 72 hours in the absence or in the presence of carnosol ranging from 6 nM to 9 μM. The experiment was repeated three times, each time using subchondral osteoblasts coming from a different donor.

### OA osteoblasts/chondrocytes co-culture

The inserts (with a pore size of 1 μm; Falcon, BD Biosciences, Belgium) containing ten alginate beads were co-cultured for 4 days with osteoblasts in monolayer, as previously described [17]. Before co-culture, osteoblasts were pre-incubated or not with carnosol for 72h. Before co-culture, osteoblasts were extensively washed with PBS. Co-culture medium was DMEM supplemented with 1% ITS+, 10 mM HEPES, 100 U/ml penicillin, 100 μg/ml streptomycin, 2 mM glutamine, 50 μg/ml ascorbic acid, 20 μg/ml proline. To identify the osteoblast-secreted mediators involved in chondrocyte responses, we have also tested piroxicam (7 μg/ml) or 5μg/ml of Mab neutralizing the activity of human IL-6, or TGF-β1 and β3 (TGFβ1/β3) or HGF (R&D systems, UK) in co-culture and monoculture. Six wells of a 12-well plate were used for each culture conditions. The experiment was repeated three times, each time using subchondral osteoblasts coming from a different donor. Conversely, the origin of OA chondrocytes was from a distinct donor than osteoblasts. As controls, OA chondrocytes in alginate beads were cultured alone (mono-culture). The cell pellets of chondrocytes were homogenized in 1 ml PBS by ultrasonic dissociation for DNA and aggrecan quantification or in 600 μl of cell lysis buffer (Qiagen) for RNA isolation. Cell extracts were kept at -80°C until analysis.

### DNA assay

The DNA content of the cell cultures was measured according to the fluorimetric method of Labarca and Paigen [18].

### LDH release assay

The cell viability was estimated by the ratio of lactate deshydrogenase (LDH) released in the culture supernatant, as previously described [16].

### Immunoassays for aggrecan, IL-6, MMP-3, TIMP-1 and PGE_2_

IL-6 and MMP-3 were directly measured in chondrocytes or osteoblasts-conditioned culture supernatant by specific EASIA (Enzyme Amplified Sensitivity ImmunoAssays, Invitrogen, Belgium). Aggrecan was measured in the different compartments of alginate bead and in culture supernatants by a specific EASIA (Invitrogen, Belgium). In the case of MMP-3, the antibodies were directed against pro-MMP-3, activated-MMP-3 and MMP-3 bound to TIMP-1 and TIMP-2. PGE_2_ was assayed according to a previously described radioimmunoassay [19].

### Alkaline phosphatase assay

Alkaline phosphatase activity was quantified in the cellular fraction of the osteoblasts culture, using p-nitrophenylphosphate as substrate, as previously described [17]. Results were expressed in nmoles of p-nitrophenol released per min and per μg of DNA.

### Nitrite assay

Nitric oxide (NO) production was determined by quantifying its derived product, nitrite, in the culture supernatant using a spectrophotometric method based upon the Griess reaction, as previously described [20]. Briefly, a sample of the supernatant or sodium nitrite (NaNO2) standard dilutions was mixed with Griess reagent (0.5% sulphanilamide, 0.05% naphtyl ethylene diamine dihydrochloride, 2.5% H3PO4). The absorption was measured at 540 nm.

### Quantitative Real-time RT PCR

RNA from 1.10^6^ cells was isolated by RNeasy mini kit (Qiagen), reverse transcription made with superscript III (Invitrogen) and polymerase chain reaction (PCR) was performed by using the Light Cycler-FastStart DNA Master Sybr Green I (LC480, Roche Diagnostics, Brussels, Belgium). The PCR template source was either 3 ng first-strand cDNA or purified DNA standard. Primer sequences used to amplify the desired cDNA were as shown in Table 1. Glyceraldehyde-3-phosphate dehydrogenase (GAPDH), a house keeping gene, was used as internal control and gene expressions were normalised by calculating the expression of aggrecan, type II collagen (COL2A1), MMP-3, ADAMTS-4, -5 and that of GAPDH.

**Table 1:** Primers Sequences .

cDNA		5’-3’	bp
GAPDH	Forward	TTGGTATCGTGGAAGGACTCA	269
	Reverse	TGTCATCATATTTGGCAGGTTT	
AGC	Forward	TCTACCTCTGCGGTAGGG	154
	Reverse	GGAAGTTCACTGACATCCTCTAT	
COL2A1	Forward	GGATGCCACACTCAAG	216
	Reverse	TTGGGGTAGACGCAAG	
MMP-3	Forward	CCCAAGAGGCATCCAC	264
	Reverse	GGGTCAAACTCCAACTGT	
ADAMTS4	Forward	AGAAGAAGTTTGACAAGTGC	225
	Reverse	GCGTGTATTCACCATTGAG	
ADAMTS5	Forward	ATCACCCAATGCCAAGG	246
	Reverse	AGCAGAGTAGGAGACAAC	

### Statistical analysis

The results (mean ± SD) were expressed as GAPDH-normalized gene expression or as the concentration per μg of DNA. A One-way Analysis of Variance (ANOVA) test followed by a Tukey-Kramer Multiple Comparisons Post-test was performed on means of all the experiments, and Pearson correlations were performed to compare concentration-dependence (GraphPad Prism 6.0). Log(agonist) or log(inhibitor) vs response non linear fit was used to estimate the IC50 (GraphPad Prism 6.0).

## Results

### Effect of carnosol on proliferation and viability of chondrocytes and osteoblasts in culture

At concentrations ranging from 6 nM to 9 μM, no significant variation of chondrocyte or osteoblast viability, as evaluated by the LDH release, was observed in our cultures. The DNA content remained stable in our culture conditions.

### Effect of carnosol on OA chondrocyte metabolism

**Fig 1:**
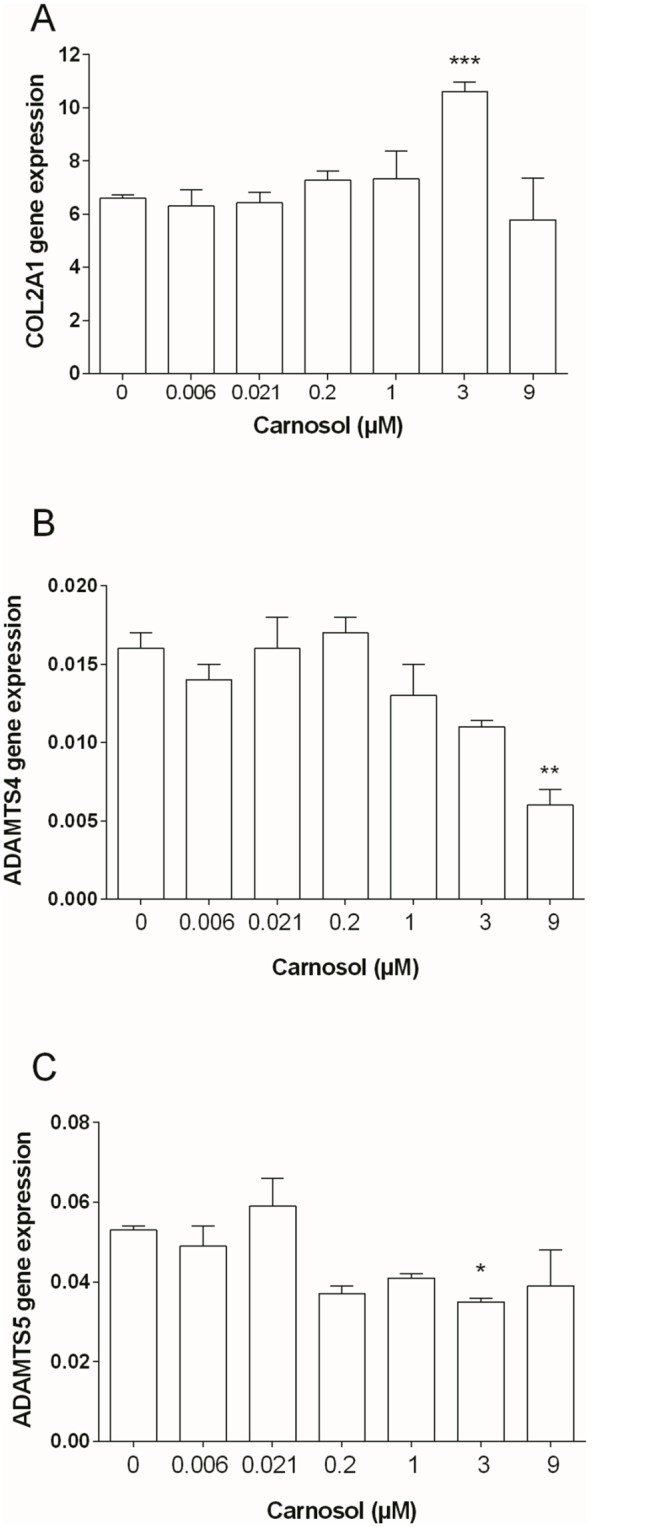
Effect of Carnosol on Chondrocyte Gene Expression. (A) COL2A1, (B) ADAMTS-4, (C) ADAMTS-5. Gene expression was normalized to GAPDH. The results are expressed as the mean ± SD of three experiments performed with cells coming from three different donors. ANOVA with Tukey posttest performed between treated and untreated cells, * = p<0.05, ** = p<0.01 and *** = p<0.001.

After 4-days of culture, carnosol did not significantly modify the aggrecan production or gene expression by chondrocytes, but significantly enhanced type II collagen gene expression at 3 μM (1.61-fold, p = 0.0008, Fig 1A). Carnosol also significantly reduced in a concentration-dependent manner the ADAMTS-4 (IC50 = 8.5 μM, r = -0.9463, p = 0.0012, Fig 1B) and ADAMTS-5 gene expression at 0.2 μM and higher concentrations (1.5-fold, p<0.05, Fig 1C).

**Fig 2:**
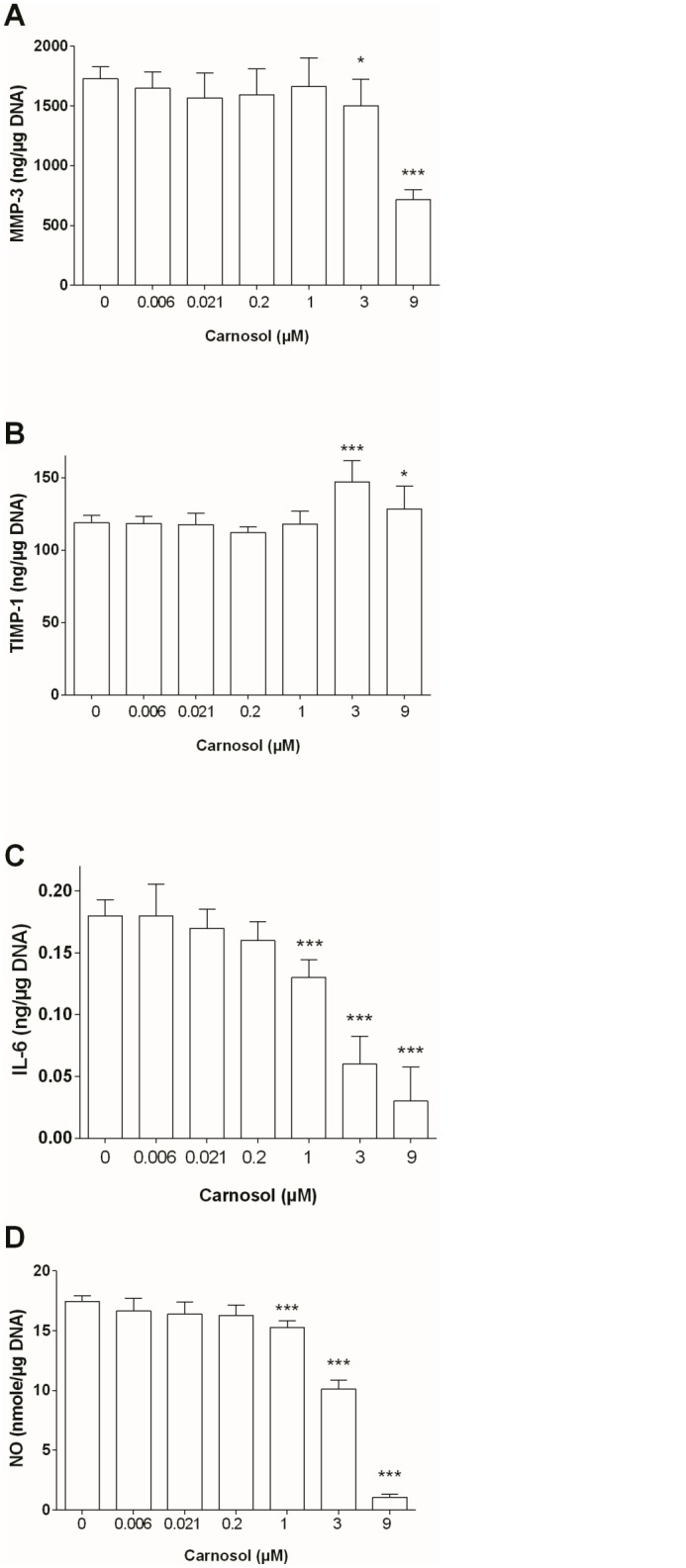
Effect of Carnosol on Chondrocyte Protein Production. (A) MMP-3, (B) TIMP-1, (C) IL-6 and (D) NO. Production was normalized to DNA content. The results are expressed as the mean ± SD of three experiments performed with cells coming from three different donors. ANOVA with Tukey posttest performed between treated and non-treated cells, * = p<0.05 and *** = p<0.001.

Carnosol significantly decreased MMP-3 protein production in a concentration-dependent manner (IC50 = ~9.6μM, r = -0.9684, p = 0.0003) and enhanced TIMP-1 protein production at 3 μM (1.24-fold, p = 0.02) by OA chondrocytes (Fig 2A and 2B). These effects were associated with a concentration-dependent decrease of the MMP3/TIMP-1 ratio (IC50 = 3μM, r = -0.9770, p = 0.0002).

Finally, carnosol significantly down-regulated chondrocyte production of IL-6 (IC50 = 2.3 μM, r = -0.9067, p = 0.0049 Fig 2C) and NO (IC50 = 5.3 μM, r = -0.9942, p<0.0001, Fig 2D) in a concentration-dependent manner. The inhibitory effect of carnosol on these parameters was statistically significant at concentration of 1 μM and above.

### Effect of carnosol on OA subchondral osteoblasts

**Fig 3:**
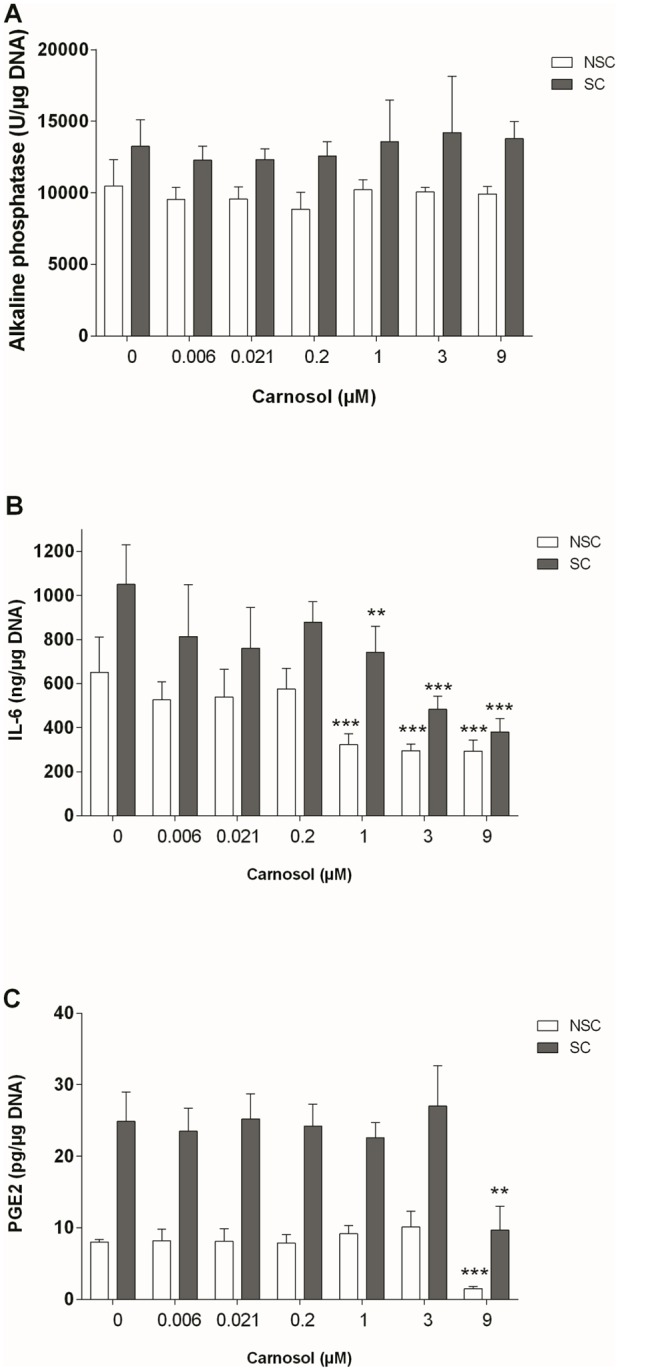
Effect of Carnosol on Activity of Alkaline Phosphatase (A), Production of IL-6 (B) and PGE_2_ (C) in NSC and SC Subchondral Osteoblasts. The results were normalized to the DNA content and are expressed as the mean ± SD of three experiments performed with cells coming from three different donors. ANOVA with Tukey posttest performed between treated and non-treated cells, ** = p<0.01 and *** = p<0.001.

Alkaline phosphatase activity was higher in SC osteoblasts than in NSC osteoblasts (10494 +/- 1833 vs 13254 +/- 1652 U/μg DNA, p = 0.006). Carnosol did not significantly affect the alkaline phosphatase activity of NSC or SC osteoblasts (Fig 3A).

SC osteoblasts produced higher levels of IL-6 and PGE_2_ than NSC osteoblasts (1.6 and 3.1-fold, p<0.001), and NO production was below detection levels in both NSC and SC osteoblasts. Carnosol reduced IL-6 production in a concentration-dependent manner (IC50 = 0.6 μM, r = -0.7674, p = 0.0440 in NSC and IC50 = 2.7μM, r = -0.7861, p = 0.0361 in SC osteoblasts, 0.45-fold at 9 μM in NSC and 0.36-fold in SC, Fig 3B). At 9 μM, carnosol inhibited PGE_2_ production in both NSC and SC cultures (0.19-fold in NSC and 0.39 fold in SC osteoblasts, p<0.01, Fig 3C).

### Effect of carnosol on OA chondrocytes co-cultured with subchondral osteoblasts

**Fig 4:**
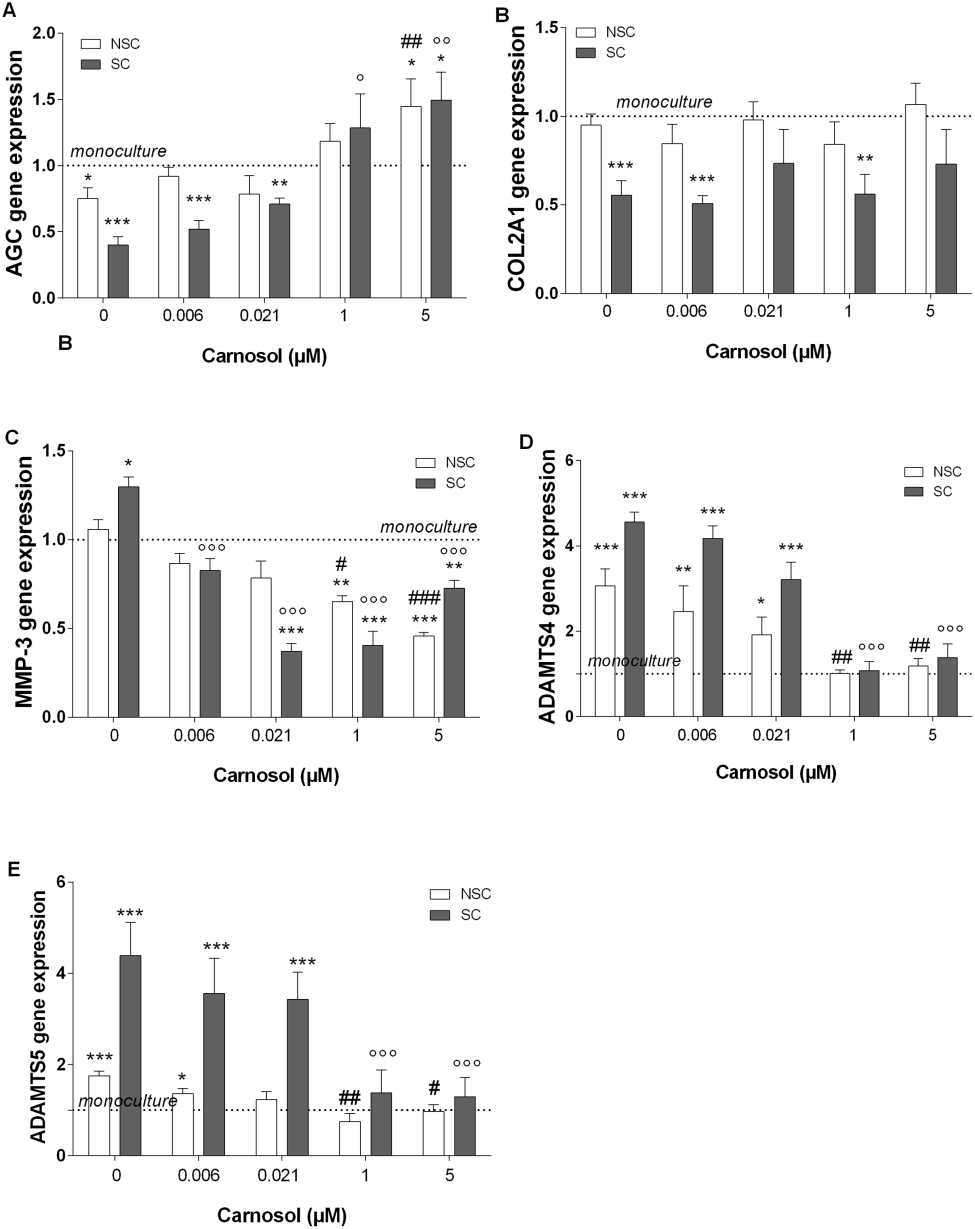
Effect of Carnosol Pre-treatment of Subchondral Osteoblasts on Chondrocyte Gene Expression after 4 Days of Co-culture with Osteoblasts. (A) AGC, (B) COL2A1, (C) MMP-3, (D) ADAMTS-4, (E) ADAMTS-5. Gene expression was normalized to GAPDH. The results are expressed as the mean ± SD of three experiments performed with cells coming from three different donors, and presented as ratio of chondrocytes monoculture experiments run in parallel. ANOVA with Tukey posttest statistical significances: Chondrocytes/osteoblasts co-culture compared to mono-culture * = p<0.05, ** = p<0.01, *** = p < 0.001; SC osteoblasts compared to NSC osteoblasts # = p<0.05, ## = p<0.01, ### = p<0.001; SC osteoblasts in co-culture with inhibitors compared to SC osteoblasts without inhibitors: ° = p<0.05, °° = p<0.01 and °°° = p<0.001.

When chondrocytes were co-cultured with osteoblasts, NSC osteoblasts did not significantly modify aggrecan protein production by chondrocytes, while SC osteoblasts significantly reduced total aggrecan production by 15+/-4% (p = 0.043). Aggrecan gene expression by chondrocytes was decreased by 25+/-8% in co-culture with NSC osteoblasts (p = 0.032) and by 58+/-7% with SC osteoblasts co-culture (p = 0.0053, Fig 4A). The pre-treatment of SC osteoblasts with carnosol at 1 or 5 μM fully prevented the inhibition of aggrecan gene expression by chondrocytes and even enhanced this gene expression by 3.7 and 4.2-fold (p<0.05 and p <0.01, Fig 4A). Type II collagen gene expression was not significantly modified in presence of NSC osteoblasts, but was reduced by SC osteoblasts (0.55-fold, p = 0.0006, Fig 4B). Carnosol did not affect the inhibitory effect of SC osteoblasts on COL2A1 gene expression (Fig 4B).

When the chondrocytes were co-cultured with NSC osteoblasts, ADAMTS-4 and -5 expression by chondrocytes were 3-fold or 1.8-fold upregulated (p<0.001), but MMP-3 gene expression was unchanged. In co-culture with SC osteoblasts, ADAMTS-4 / -5 and MMP-3, mRNA levels were 4.6-fold, 4.4-fold (p<0.001) and 1.3-fold (p<0.05), respectively, increased compared to chondrocytes in mono-culture. Pre-incubation of SC or NSC osteoblasts with carnosol at 1 or 5 μg/ml induced a significant reduction of ADAMTS-4 / -5 and MMP-3 gene expression by chondrocytes (SC: 0.3-fold, p<0.001, NSC: 0.5-fold, p<0.05, Fig 4C–4E).

### Effects of subchondral sclerotic osteoblast mediators on chondrocyte

**Fig 5:**
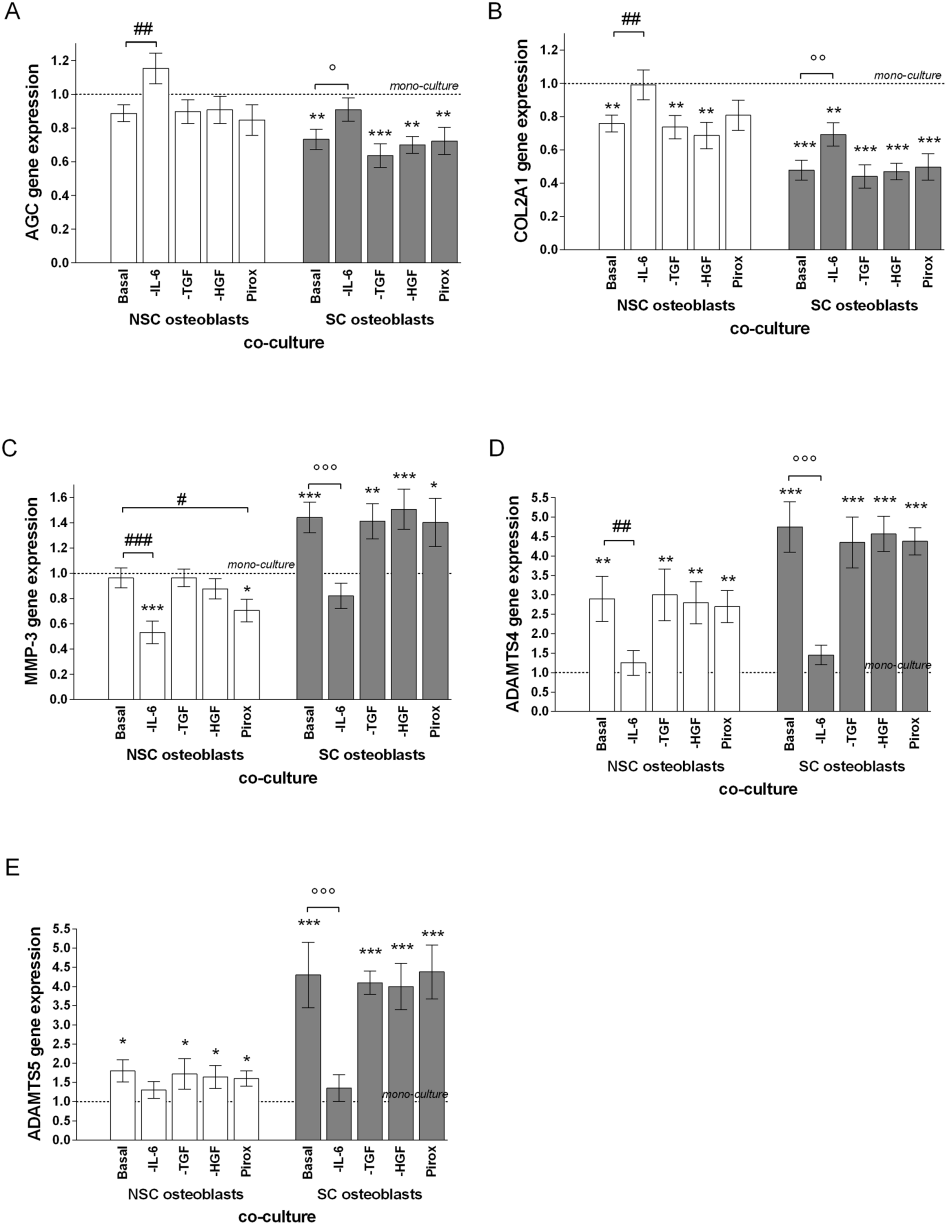
Effect of IL-6 Neutralizing Mab on Gene Expression by Chondrocytes. (A) AGC, (B) COL2A1, (C) MMP-3, (D) ADAMTS-4 and (E) -5 gene expressions by chondrocytes after four days of culture in the absence (mono-culture) or in the presence (co-culture) of osteoblasts isolated from sclerotic (SC) or non-sclerotic (NSC) zones of subchondral bone. The culture was performed in absence or in the presence of 5 μg/ml of anti-IL-6, TGF-β1/3 or HGF Mab or 7 μg/ml of piroxicam (Pirox). GAPDH-normalized gene expressions are reported to mono-culture expression level, and represented as the mean ± SD of three experiments performed with cells coming from three different donors. Comparison of mean values of gene expression was performed by Mann-Whitney tests. Statistical significances: Chondrocytes/osteoblasts co-culture compared to mono-culture * = p<0.05, ** = p<0.01, *** = p < 0.001; SC osteoblasts compared to NSC osteoblasts # = p<0.05, ## = p<0.01, ### = p<0.001; SC osteoblasts in co-culture with inhibitors compared to SC osteoblasts without inhibitors: ° = p<0.05 °° = p<0.01 and °°° = p<0.001.

To identify the osteoblastic mediators triggering the chondrocytes responses, we incubated subchondral osteoblast/chondrocyte co-cultures in the presence of monoclonal antibodies (Mabs) neutralizing either IL-6 or TGF-β1 or HGF activity, or with piroxicam as PGE_2_ inhibitor (Fig 5). The addition of the Mabs or piroxicam completely neutralized the expression of these proinflammatory mediators as of IL-6, TGF-β1 or PGE_2_ by were non detectable in the culture supernatants by specific ELISA tests. In comparison to chondrocyte mono-culture with the same neutralizing agents, only anti-IL-6 Mab significantly inhibited the effect of SC osteoblasts on AGC (1.45-fold, p = 0.0009), COL2A1 (1.8-fold, p = 0.0312), MMP-3 (0.5-fold, p = 0.0008), ADAMTS-4 (0.29-fold, p<0.0001) and -5 (0.29-fold, p<0.0001) gene expression (Fig 5). The same range of effect was observed also on NSC osteoblasts (Fig 5). Piroxicam also significantly decreased the MMP-3 expression (p = 0.03) in NSC osteoblasts co-culture.

## Discussion

In this study, we have shown that *in vitro*, at similar ranges of concentrations to those bioavailable in tissues after rosemary extracts administration [21], carnosol is a good candidate as anti-OA agent. On chondrocytes, carnosol has an interesting stimulating effect on type II collagen gene expression and an inhibitory effect on MMP-3 production, and ADAMTS-4 and -5 gene expression. Further, TIMP-1 synthesis was increased by carnosol suggesting that this product can also down-regulate MMP activity. Carnosol showed also potent anti-inflammatory activities by decreasing NO and IL-6 synthesis.

In addition to these direct effects on chondrocyte metabolism, carnosol also exerts a protective effect on the deleterious activities of OA subchondral osteoblasts on chondrocytes, observed in our co-culture model. Indeed, pre-treatment of OA sclerotic subchondral osteoblasts with carnosol mediated an increase in cartilage matrix formation components (aggrecan, type II collagen) and decrease in gene expression of degradation enzymes (MMP-3, ADAMTS4 and -5) by chondrocytes.

As previously shown by ourselves [17, 22] and others [23, 24], OA subchondral osteoblasts, in particular coming from sclerotic subchondral bone, can modify the gene expression of chondrocytes in co-culture, leading to a phenotype suitable for matrix degradation We therefore tested the effect of carnosol on certain factors in OA subchondral osteoblasts which could be responsible for mediating the protective effects of carnosol on matrix formation and degradation in cocultures. Carnosol did not modify alkaline phosphatase activity of the osteoblasts, but did it reduce IL-6 production at 1 μM, and at 9 μM also PGE_2_ production. These effects of carnosol were observed in the same way either on NSC and SC osteoblasts.

Finally, we investigated the effects of cytokines and growth factors involved in bone remodelling for their potency to trigger the deleterious effects of SC osteoblasts on the overlying cartilage in OA. TGF-β1/3, IL-6 and PGE_2_ are regulators of bone remodelling and are involved in subchondral bone sclerosis associated with OA. Moreover, they are overexpressed in OA sclerotic subchondral bone [5, 25, 26]. Further, HGF, a factor produced by subchondral osteoblasts, but not by chondrocytes, was found in the deep layer of the cartilage indicating that factors produced by bone cells can cross over the osteochondral bone plate [27]. In our co-culture model, we tested the effects of neutralizing factors to each of these mediators, and among them only IL-6 seems to be involved. To the best of our knowledge, this is the first time that IL-6 is identified as a key mediator of the cross talk between subchondral bone and cartilage in OA.

We observed that the inhibitory effect of SC osteoblasts on the production of aggrecan by chondrocytes was abolished by neutralizing the biological activity of IL-6. In addition, type II collagen gene expression inhibition by osteoblasts was reversed by about 50% in the presence of anti-IL-6. Neutralization of IL-6 also abolished the effect of SC osteoblasts on the expression of MMP-3, ADAMTS-4 and -5 by chondrocytes. Previously, Sanchez et al. [28] demonstrated that IL-6 in the presence of its soluble receptor, stimulated the production of MMP-3 and inhibited the synthesis of aggrecan by chondrocytes in alginate beads. Other authors have shown that IL-6 inhibited the expression of type II collagen and the aggrecan link protein by bovine chondrocytes cultured in monolayer, in parallel with an inhibition of the expression of the transcription factor sox9 [29]. IL-6 acts in synergy with IL-1 in vitro to degrade collagens and proteoglycans in cartilage explants [30]. These results emphasize the key role played by IL-6 in cartilage degradation. They also emphasize the important role of this cytokine in the crosstalk between subchondral bone and cartilage. Furthermore, these results are consistant with previous results showing that pre-treatment of NSC osteoblasts with IL-6 led to the same deleterious effect than untreated SC osteoblasts on chondrocytes in co-culture [17]. Finally, these data indicate that the protective effects of carnosol against osteoblast-induced deregulation of chondrocyte metabolism is probably mediated by IL-6.

While we cannot exclude the involvement of other factors, our study clearly demonstrates that PGE_2_ TGF-β1–3 and HGF are not involved in the cross-talk between osteoblasts and chondrocytes, and identifies IL-6 as a key factor of the osteoblasts/chondrocytes crosstalk in OA.

## Conclusion

Carnosol showed potent anti-inflammatory and anti-catabolic effects on chondrocytes. Furthermore, it was able to reduce cartilage matrix breakdown and enhance its formation in presence of subchondral osteoblasts, probably by inhibition of IL-6 production by osteoblasts. Its effects would deserve to be further confirmed and investigated, in an appropriate animal model and then in clinical studies.
